# The association between tooth loss and serum cholesterol levels in adults: a scoping review

**DOI:** 10.1186/s12903-025-07431-y

**Published:** 2025-12-06

**Authors:** Keyi Wan, Kexin Wan

**Affiliations:** 1 Shanghai Jiao Tong University School of Medicine, Shanghai, China; 2 Peking University Health Science Center, Beijing, China

**Keywords:** Tooth loss, Cholesterol, Dyslipidemia, Edentulism, Masticatory function

## Abstract

**Objective:**

This scoping review systematically mapped the evidence on the association between tooth loss and serum cholesterol levels in adults, addressing a significant gap in understanding the direct link beyond broader systemic conditions such as cardiovascular disease.

**Methods:**

In accordance with the PRISMA-ScR guidelines, a comprehensive search of four electronic databases (PubMed, MEDLINE, EMBASE, and Cochrane Library) was conducted from July 1968 to September 2025. Studies involving adults (≥ 18 years) that quantified tooth loss and reported serum lipid profiles specifically high-density lipoprotein cholesterol, low-density lipoprotein cholesterol, total cholesterol, and/or triglycerides levels — were included.

**Results:**

Among the 23 studies synthesized, a majority demonstrated a significant association between greater tooth loss and adverse lipid profiles, characterized by lower high-density lipoprotein cholesterol levels, higher low-density lipoprotein cholesterol levels, and elevated triglyceride levels. A dose-response relationship was evident, with more extensive tooth loss correlated with progressively worsening cholesterol levels. Analysis of edentulous populations suggested that the primary mechanism in this population may be nutritional maladaptation from impaired masticatory function rather than systemic inflammation. Some evidence indicates a bidirectional relationship, where dyslipidemia may also be a risk factor for tooth loss. Notably, prosthetic rehabilitation for partial tooth loss was associated with improved cholesterol levels, an effect not consistently observed with complete dentures.

**Conclusion:**

This scoping review suggests an association between tooth loss and dyslipidemia, which may be mediated through dietary changes due to reduced masticatory function and systemic inflammatory pathways. However, as a scoping review that included studies of heterogeneous designs and did not perform a formal risk of bias assessment, these results are indicative of an association and cannot establish causality. Despite these limitations, the gathered evidence underscores the importance of considering oral health when developing strategies to maintain metabolic health.

## Background

The increasing global burden of tooth loss has positioned it as a critical public health priority, with epidemiological surveillance revealing that severe tooth loss [1, 2] (defined as ≥ 8 missing teeth) affects people worldwide. According to the 2024 Oral Health Surveillance Report of the U.S. Centers for Disease Control and Prevention (CDC), the mean number of permanent teeth decreases from 27.0 among adults aged 20–34 to 19.8 among those aged 75 or older. Concurrently, the prevalence of edentulism increases from 1.2% (ages 35–49) to 19.7% (ages 75+), indicating that nearly one-fifth of the oldest adults have lost all their teeth [3]. Significant inequalities are evident, with notably lower mean numbers of teeth among non-Hispanic Black individuals (16.2), current smokers (16.3), adults with less than a high school education (16.8), and those in middle-to-high poverty groups (17.6) [3]. As the terminal sequela of progressive periodontitis or trauma, tooth loss exceeds mere dental compromise—it embodies a confluence of chronic oral inflammation and irreversible masticatory functional impairment.

In addition to oral health concerns, dyslipidemia—encompassing abnormal serum levels of total cholesterol (TC), triglycerides (TG), low-density lipoprotein cholesterol (LDL-C), and high-density lipoprotein cholesterol (HDL-C)—remains a cornerstone of metabolic disease pathogenesis. The global prevalence rates were reported to be 28.8% for hypertriglyceridemia, 24.1% for hypercholesterolemia, 38.4% for low HDL-C, and 18.93% for high LDL-C. Hypertriglyceridemia was more common in men (33.8%) than in women (24.5%), whereas low HDL-C was more frequent in women (40.5%) than in men (34.1%). The highest rates of low HDL-C and mixed dyslipidemia were observed in the Middle East and Latin America [4]. Critically, cholesterol aberrations manifest decades before clinical endpoints, offering a modifiable early window for intervention [5].

Current studies focus mainly on two pathways: (1) The relationship between oral inflammation (e.g., periodontitis) and altered serum cholesterol levels, proposing mechanisms such as systemic inflammation promoting hepatic lipid synthesis [6–8]. (2) Relationships between the number of missing teeth and common diseases (e.g., cardiovascular disease (CVD) and metabolic syndrome (MetS)). These findings demonstrated that a greater number of missing teeth is correlated with an increased incidence of coronary heart disease and stroke [9].

Despite these advances, an obvious knowledge gap persists: direct evidence quantifying tooth loss-cholesterol associations remains limited. While cholesterol is a plausible mediator of the relationship between tooth loss and CVD, no comprehensive synthesis exists to quantify this connection or address confounding factors like methodological heterogeneity in the definition of tooth loss. This omission is particularly notable given cholesterol’s role as an overall health indicator that may reveal early, modifiable risks across multiple disease states.

To resolve the mechanistic ambiguity behind this association, our review accords primacy to edentulous populations—a cohort representing a natural experiment. Complete tooth loss typically eliminates active periodontal inflammation through the absence of gingival sulci and periodontal ligaments. Therefore, cholesterol abnormalities in edentulous individuals likely reflect nutrient maladaptation rather than inflammatory cascades, offering a novel lens to investigate oral function-metabolism crosstalk.

This scoping review therefore synthesized 23 studies to map the direct association between the degree of tooth loss and serum cholesterol levels. We investigated how classification methods (dichotomous dentate/edentulous vs. multiclass tooth-count strata) influence the observed effects, whether comorbidities like diabetes or MetS modify associations; and how longitudinal tooth loss progression reshapes dyslipidemia trajectories. We also discussed whether tooth loss independently predicts dyslipidemia beyond inflammatory mediators. Furthermore, our findings informed prosthodontic interventions targeting metabolic health—such as optimizing masticatory function to facilitate changes in serum cholesterol levels.

## Methods

This scoping review follows the PRISMA-ScR reporting guidelines. The protocol was registered on the Open Science Framework (Registration DOI: 10.17605/OSF.IO/7FUA9).

### Search strategy

A comprehensive literature search was conducted across four electronic databases (PubMed, MEDLINE, EMBASE, and Cochrane Library) from July 1968 to September 2025, without language restrictions. The search strategy combined key terms related to tooth loss (“tooth loss,” “edentulous,” “edentulism”) and cholesterol markers (“cholesterol,” “HDL,” “LDL,” “TC,” “lipoprotein,” “CVD,” “atherosclerosis,” “metabolic syndrome,” “MetS”) using Boolean operators. The Boolean operators and search terms, are provided in Appendix. To ensure robustness, the strategy was peer-reviewed by an independent dental doctor. Additional records were identified through manual screening of reference lists from relevant reviews.

### Eligibility criteria

Studies were included if they:

1. Involved adults (≥18 years), regardless of health status (e.g. CVD,diabetes).
2. Quantified tooth loss (e.g., dichotomized as edentulous versus dentate, or multiclass groupings on the basis of the remaining teeth).
3. Reported serum cholesterol outcomes (TC, HDL-C, LDL-C and/or TG). All study designs were considered (cross-sectional, case-control, cohort, reviews, etc.), but editorials and nonhuman studies were excluded.

### Study screening and selection

The search results were deduplicated using EndNote 20. Two independent reviewers (Keyi W. and Kexin W.) screened titles and abstracts against the eligibility criteria respectively, followed by full-text assessment of potentially relevant articles. Any discrepancies between the two reviewers were resolved through a consensus meeting. In cases where consensus could not be reached, a third reviewer was consulted for adjudication.

**Fig. 1:**
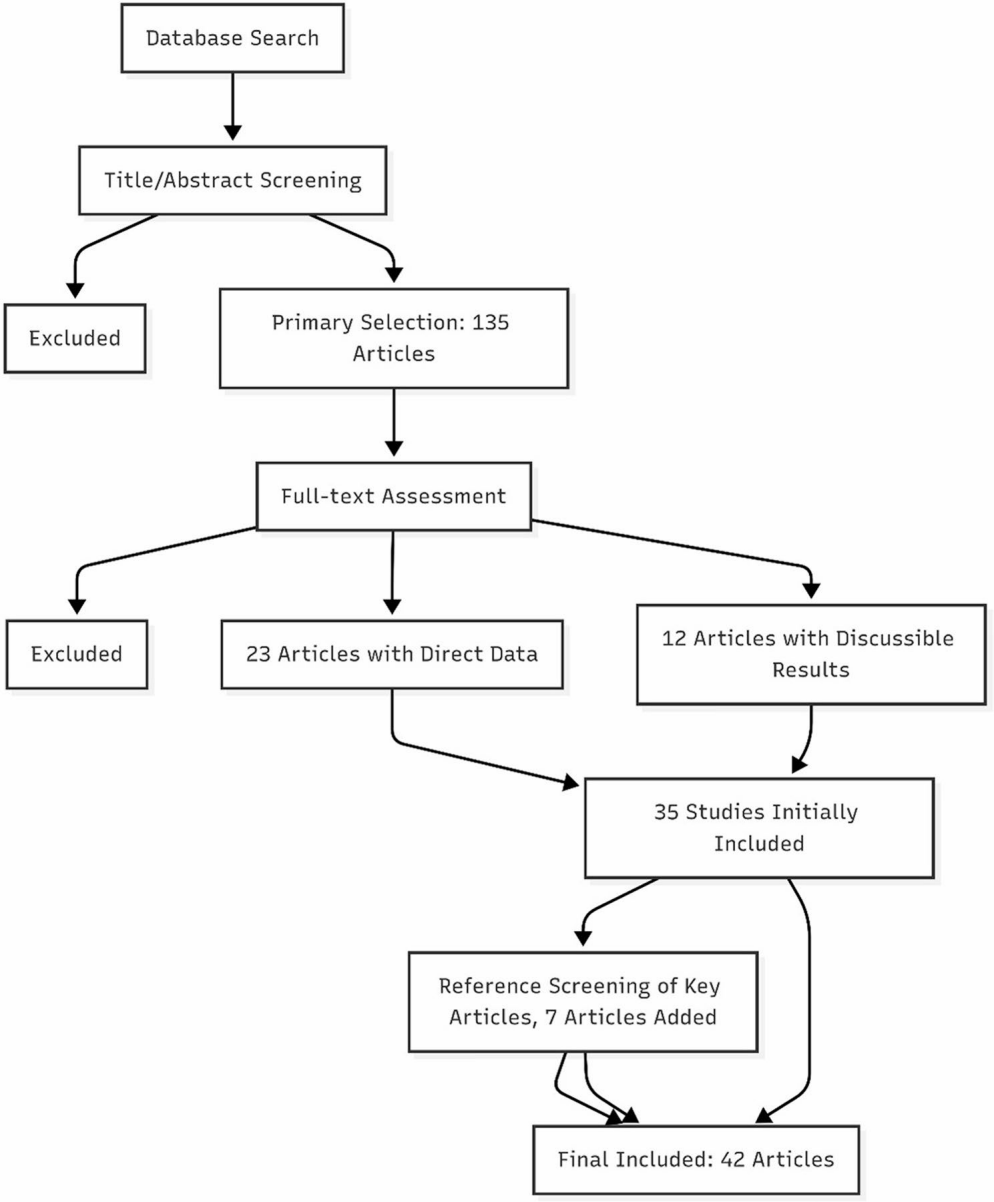
PRISMA flow diagram of the study selection process

The selection process was documented using a PRISMA flow diagram (Fig. 1).

### Consideration of study quality and risk of bias

In accordance with the primary objective of a scoping review—to map the scope, concepts, and types of available evidence rather than to appraise the quality of evidence or synthesize findings—a formal risk of bias or quality assessment of the included studies was not performed. This methodological approach is consistent with the PRISMA-ScR guidelines, which emphasize the mapping of literature regardless of its methodological rigor.

### Data extraction

An extraction table (Table 1) was developed to analyze the 23 key studies.

**Table 1:** Detailed information of the 23 key studies

Group	Title	Author, year of publication	Study design	Aim	Setting	Number (male, female)	Health conditions	Tooth loss classification	Tooth loss measurement methodology	Cholesterol measurement methodology	Edentulous group	Key confounders adjusted	Conclusion
Group 1	Associations of Periodontal Damage and Tooth Loss with Atherogenic Factors among Patients with Type 2 Diabetes Mellitus	Furukawa et al. (2007) [10]	Cross-sectional study	To clarify the associations of periodontal dmage and tooth loss with atherogenic factors (e.g., cholesterol) in type 2 DM patients	Outpatient clinics (Aichi Medical University Hospital and Fushimi Clinic, Japan)	100 adults (72%M, 28%F)	Participants have DM2, no participants with DM1, or CVD, or metabolic syndrome at enrollment	≥20 teeth vs. <20 teeth	Number of remaining teeth (third molars excluded). Implants and dentures were excluded. Clinical dental examination counting decayed, missing, and filled teeth.	Measured via blood analysis	No	Age, sex, smoking, oral hygiene score, HbA1c, , mean depth of periodontal pockets, risk factors for periodontal disease, BMI, GFR, BP	1. HDL-C positively associated with number of teeth2. TC no significant association with tooth loss
	Coffee Intake as a Risk Indicator for Tooth Loss in Korean Adults	Song et al. (2018) [11]	Cross-sectional study	To examine the association between coffee intake and tooth loss in Korean adults	South Korea (KNHANES 2010–2011)	7299 adults (48.3%M, 46.4%F)	General population	Number of remaining teeth (grouped as ≥20 or <20)	Not mentioned	Not mentioned	No	Not mentioned	Participants with <20 teeth had higher LDL-C
	Factors Related to Tooth Loss Among Community-Dwelling Middle-aged and Elderly Japanese Men	Ando A. et al. (2013) [12]	Cross-sectional study	To identify factors associated with tooth loss in Japanese men aged 40–79 years	Community-based health check-ups in Northern Japan (Iwate Prefecture, 2002–2005).	8352adults (M)	General community-dwelling men	Self-reported teeth count: 0, 1–9, 10–19, ≥20 teeth.Analysis grouped as ≤19 teeth vs. ≥20 teeth	Self-reported (Self-administered questionnaire: "How many teeth do you have?) teeth/dentures/implants	HDL-C: Measured enzymaticallyTC: Measured enzymatically (standardized by CRMLN).	Yes: 1764 men (21.1% of the cohort)	Age, BMI, SBP, albumin, log-transformed hsCRP, smoking status (current smoker, ex-smoker, or non-smoker), alcohol drinking status (current drinker or not), marital status (single or not), and education level (low or not)	1. Age 40–64 yrs: Decreased HDL-C significantly associated with increased tooth loss2. Age 65–79 yrs: No significant association
	Tooth Loss and Carotid Intima-Media Thickness in Relation to Functional Atherosclerosis: A Cross-Sectional Study.	Shimizu Y. et al. (2022) [13]	Cross-sectional study	To investigate the association between structural atherosclerosis (CIMT) and tooth loss, stratified by functional atherosclerosis (CAVI) status	Goto City, Japan (community-based health check-ups, 2016-2018)	1235 adults (39%M, 61%F)	General community-dwelling adults; excluded edentulous participants	Men: >20 or≤20 remaining teethWomen: >19 or≤19 remaining teeth	Natural teeth only; excluded third molars. Dentures/implants not specified.	Not mentioned	No	Age, sex	Higher HDL linked with fewer remaining teeth
	Tooth Loss Related with Prevalence of Metabolic Syndrome in a General Urban Japanese Population: The Suita Study	Ono et al. (2022) [14]	Cross-sectional within a longitudinal cohort	To identify whether ≤19 teeth serves as a surrogate marker for metabolic syndrome (MetS) risk	Japan Suita	3771 adults (45%M, 55%W)	Not mentioned	≥20 teeth or≤19 teeth​	Self-reported tooth count validated by dental exams (92% concordance rate)	Not mentioned	No	Age, current smoking, current drinking and with/without medical history	1. HDL-C is significantly lower in ≤19 teeth group2. TG is higher in ≤19 teeth group3. LDL-C: No significant association
	Dental Status, Diet and Cardiovascular Risk Factors in Middle-Aged People in Northern Sweden	Johansson et al. (1994) [15]	Cross-sectional analysis	To compare the dietary intake and the levels of traditional cardiovascular (CVD) risk factors in edentulous middle-aged individuals and individuals of the same age and sex who still had natural teeth	Northern Sweden (Norrbotten & Västerbotten)	2617 adults (49%M, 51%F)	General population	Edentulous (full dentures in both jaws) vs. dentate (natural teeth with no dentures)	1. Do you only have your natural teeth?2. Do you have natural teeth and a removable partial denture?3. Do you wear full dentures?	Enzymatic methods (commercial kits)	Yes	Age, sex	1. HDL-C significantly decreases in edentulous vs. dentate2. TC increeases in edentulous women (p<0.001), but not in men3. TG increases in edentulous women (p<0.001), but not in men
	Complete Tooth Loss and Allostatic Load Changes Later in Life: A 12-Year Follow-Up Analysis of the English Longitudinal Study of Ageing	de Oliveira et al. (2021) [16]	Longitudinal (12-year follow-up)	1) Association between complete tooth loss (edentulism) and 12-year AL changes.2) Role of fruit/vegetable consumption.	English Longitudinal Study of Ageing (ELSA), UK	2430 adults (45%F, 55%M)	Community-dwelling older adults	Dentate (≥1 natural tooth) vs. Edentulous ( Complete tooth loss)	self-reported edentulism (no natural teeth)	Not mentioned	Yes	Education, wealth quintiles, smoking status, self-reported physical activity, fruit and vegetable consumption	1. Edentulism linked to lower HDL over 12 years2. Edentulous participants showed a faster decline in HDL over 12 years3. No significant association linked to LDL and triglycerides
	Oral health and changes in lipid profile: A nationwide cohort study	Song et al. (2022) [17]	Longitudinal Cohort Study	To investigate the association of periodontitis and oral hygiene indicators with longitudinal changes in blood lipid parameters	South Korea (NHIS-HEALS database)	65078 adults (62.8%M, 37.2%F)	General population; excluded those on lipid-lowering drugs	presence of tooth loss vs. absence of tooth loss	Not mentioned	Measured using a >6-hour fasting venous blood sample	No	Sex, age, household income, smoking, alcohol, exercise, body mass index, hypertension, diabetes mellitus, chronic kidney disease, aspartate aminotransferase level, alanine aminotransferase level, baseline lipid level, markers for oral health status, and oral hygiene care	1. The presence of tooth loss was significantly associated with an increase in TG levels over time2. A paradoxical finding showed tooth loss was associated with a slight decrease in LDL-C3. No association was found between tooth loss and HDL-C
	Type 1 diabetes and oral health: Findings from the Epidemiology of Diabetes Interventions and Complications (EDIC) study	Steigmann et al. (2022) [18]	Longitudinal Cohort Study	To describe oral health outcomes and examine risk factors for tooth loss in adults with long-standing type 1 diabetes (T1 DM)	Multicenter study, USA & Canada (DCCT/EDIC cohort)	950 (50.6%F, 49.4%M)	All participants have long-standing T1 DM (mean duration: 34.6 yrs).	presence of tooth loss vs. absence of tooth loss	Not mentioned	Not mentioned	No	Age and current tobacco use	Higher HDL/LDL ratio → Decreased odds of tooth loss
Group 2	Missing teeth predict incident cardiovascular events, diabetes, and death	Liljestrand et al. (2015) [19]	Prospective cohort study (13 year follow-up)	To give predictive value of tooth loss for chronic diseases and mortality	Nationwide Finnish population-based cohort.	8446 adults (45.3%M, 54.7%F)	Some participants have diabetes, HTN, inflammation	stratified by tooth loss: 0–1, 2–4, 5–8, 9–31, edentulous	Clinical count of remaining teeth by trained nurses (baseline)	Not mentioned	Yes	Age, sex, and education years	1. HDL is inversely associated with tooth loss2. TG is significantly associated with more missing teeth3. TC is not related with tooth loss
	The association between tooth loss and cognitive decline in the aged population: The mediating role of HDL-cholesterol	Chen et al. (2023) [20]	Cross-sectional study	To investigate HDL-C as a mediator between tooth loss and cognitive decline	Information from NHANES database (USA)	1124 adults (50.1%M, 49.9%F)	Community-dwelling older adults	stratified as ≥20 teeth (control), 1-19 teeth (moderate), 0 teeth (severe)	Wisdom teeth, implants, dentures not explicitly addressed	Not mentioned	Yes, 203 participants (18.1%) were classified in the severe tooth loss Group (edentulous)	Age, BMI, SBP, DBP, sex, alcohol intake, smoking habits, race, family income, depression, marital status, education, diabetes	Severe tooth loss is associated with lower HDL-C
	Association Between Number of Missing Teeth and Hyperlipidemia: The TCLSIH Cohort Study	Qiao et al. (2024) [21]	Prospective cohort study (median follow-up: 4.2 years)	To investigate sex-specific associations between tooth loss and incident hyperlipidemia	China	13932 adults (49.6%M, 50.4%F)	Not mentioned	0 missing teeth,1–2 missing teeth​,≥3 missing teeth	Self-reported counts validated against dental exams (92% concordance rate)(≥3 missing teeth indicating ​chronic periodontitis history​ or severe caries)	Via blood test	No	Age, BMI, smoking status, family history, disease (CVD, hypertension, hyperlipidemia, diabetes), drinking status, hypertension, education level, depressive symptoms, working status, healthy pattern score, total energy intake, household income, physical activity, sweet pattern score, animal pattern score, baseline of serum lipids including LDL, TG and HDL	1. TG increases in participants with tooth loss (p<0.001)2. LDL same as above3. HDL not significant
	Associations of Number of Teeth with Risks for All-Cause and Cause-Specific Mortality in Middle-Aged and Elderly Japanese Men: The Iwate-KENCO Study	Ando A. et al. (2014) [22]	Prospective cohort study (5.6 year follow-up)	To determine associations between tooth count and all-cause/cause-specific mortality in Japanese men	Northern Japan (Iwate Prefecture; 17 municipalities, 2002–2009).	7779 adults (M)	CVD-free at baseline (no stroke or MI)	0, 1–9, 10–19, ≥20 missing teeth	Self-reported (Self-administered questionnaire: "How many teeth do you have?) teeth/dentures/implants	HDL-C: Measured enzymaticallyTotal Cholesterol (TC): Measured enzymatically (standardized by CRMLN).	Yes, total with 0 teeth: 1,764 men (21.1% of the cohort)	The study was not designed to investigate the relationship between cholesterol and tooth loss, so no confounders were specifically adjusted for in the context of this specific association	1. The Edentulus group has the lowest TC and HDL2. The mean TC is decreased with fewer remaining teeth3. Men with more than 20 teeth had the highest HDL-C, with those with 10-19 had the lowest
	The Number of Teeth is Inversely Associated With Metabolic Syndrome: A Korean Nationwide Population-Based Study	Hye-Sun Shin et al. (2017)	Cross-sectional study	To evaluate the association between the number of natural teeth and metabolic syndrome (MetS)	South Korea (KNHANES 2012–2014)	13066 adults (5758M, 7308F)	General population; included those with and without MetS, diabetes, etc.	28, 20–27, 0–19 remaining teeth	Number of existing permanent teeth (excluding wisdom teeth, implants, impacted teeth)	Not mentioned	No	Age, gender, income, education, tooth-brushing frequency, periodontitis, smoking, drinking, physical activity, and diabetes mellitus	1. Inverse association between the number of teeth and reduced HDL cholesterol2. No significant association with triglycerides after full adjustment
	Associations Between the Number of Natural Teeth and Metabolic Syndrome in Adults	Zhu & Hollis (2015) [24]	Cross-sectional study	To explore associations between number of natural teeth and metabolic syndrome	US (NHANES 2005–2008)	5511 adults (gender ratio not mentioned)	General population, some with metabolic syndrome, diabetes, or CVD	full dentition (28 teeth), 21–27 teeth, 1–20 teeth, edentulous	Excluded third molars; functional dentition defined as ≥21 teeth	By an automated enzymatic analyzer (Hitachi 7600; Hitachi, Ltd., Tokyo, Japan)	Yes	Age, sex, race, ratio of family income to poverty, physical activity, smoking status, and energy intake	A significant positive association between the number of natural teeth and serum HDL cholesterol concentration
	Number of Teeth and Selected Cardiovascular Risk Factors Among Elderly People	Syrjälä et al. (2010) [25]	Cross-sectional study	To investigate the association between the number of teeth and selected cardiovascular risk factors in an elderly population	Kuopio, Finland (Population-based Kuopio 75+ study)	523 adults (27.3%M, 72.7%F)	General community-dwelling elderly population	missing teeth: 0 teeth, 1-9 teeth, 10-19 teeth, 20-29 teeth	Not mentioned	Not mentioned	No	Sex, age, basic education, diabetes, physical ability	Fewer teeth → Lower HDL-C, higher TG, vice versa
Group 3	Relationship Between Oral Health, Diabetes Management and Sleep Apnea	Cinar et al. (2013) [26]	Cross-sectional study	To assess links between tooth loss, oral behavior, DM2, obesity, and sleep apnea.	Outpatient clinics in Istanbul, Turkey	165 adults (38%M, 62%F)	Type 2 diabetes (DM2)	dichotomization at the mean: based on maxillary (4.49 teeth lost), mandibular (3.12) and total (7.92)	No wisdom, implants, denture details	Blood tests	No	not mentioned	Tooth loss itself showed no direct correlation with HDL/LDL levels
	Tooth Loss and Metabolic Syndrome in Middle-Aged Japanese Adults	Furuta et al. (2016) [27]	retrospective longitudinal study	To examine the association between MetS and tooth loss over 5 years	Workplace health check-ups (Yokohama, Japan)	2107 adults (1718M, 389F)	Not mentioned	presence of tooth loss vs. absence of tooth loss	Clinical dental exams (tooth count), tooth loss defined as loss of ≥1 tooth over 5 years	Via blood test	No	mean pocket depth, age, gender, smoking, occupational status, DMFT, oraly hygiene status, number of teeth, tooth brushing frequency	HDL not significantly associated with tooth loss
	Tooth Loss Is Associated With an Increased Risk of Hypertension in Postmenopausal Women	Taguchi et al. (2004) [28]	Cross-sectional study	To investigate if tooth loss is associated with an increased risk of hypertension in postmenopausal women	Hiroshima University, Japan	98 adults (F)	Postmenopausal women, healthy, non-smoking, non-diabetic, no clinical atherosclerosis	presence of tooth loss vs. absence of tooth loss	Not mentioned	Routine chemical methods (Freidewald method)	No	the study was not designed to investigate the relationship between cholesterol and tooth loss, so no confounders were specifically adjusted for in the context of this specific association	No significant association found between tooth loss and any cholesterol measure (Total-C, HDL-C, LDL-C, TG)
	Total Tooth Loss and Prevalent Cardiovascular Disease in Men and Women: Possible Roles of Citrus Fruit Consumption, Vitamin C, and Inflammatory and Thrombotic Variables	Lowe et al. (2003) [29]	Cross-sectional study	To investigate mechanisms linking edentulism to CVD	North Glasgow (MONICA Project, 1992)	1269 adults (605M, 664F)	Some have CVD (35%M, 34%F)	edentulous vs. dentate	Edentulous: Complete absence of natural teeth (with or without dentures), dentate is presence of at least one natural tooth	Not mentioned	Yes	age, social class, and smoking status	Cholesterol levels were nearly identical between edentulous and dentate groups
Group 4	Relationship Between Periodontal Disease, Tooth Loss, and Carotid Artery Plaque	Desvarieux et al. (2003) [30]	Cross-sectional analysis within a prospective cohort	To investigate relationships between periodontal disease, tooth loss, and subclinical atherosclerosis	Northern Manhattan, NY (community-based)	711 adults (42%M, 58%F)	No history of stroke/MI; excluded chronic inflammatory diseases	missing teeth: 0–9, 10–19, 20–31, edentulous	Excluded wisdom teeth; dentate/edentulous status recorded	Not mentioned	Yes	age, sex	No significant differences in total cholesterol, LDL-C, or HDL-C across tooth loss categories
Group 5	Nutritional Assessment of Denture Wearers Using Matched Electronic Dental-Health Record Data	Gomez et al. (2022) [31]	Retrospective cohort study	To assess the nutritional status of denture wearers using laboratory biomarkers	Indiana University dental clinics (2010–2018)	10481 adults (45%M, 55%F)	General adult population; excluded chronic inflammatory diseases	Completely edentulous, partially edentulous, single-arch edentulism	Defined by prosthodontic treatments (CDT codes); excluded fixed prosthesis-only patients	Not mentioned	Yes	age, sex	1. No significant changes in cholesterol biomarkers post-denture placement2. No association between tooth loss (via denture use) and serum cholesterol levels
	Serum Lipoprotein Subfractions are Associated with the Periodontal Status: Results from the Population-Based Cohort SHIP-TREND	Kapp et al. (2024) [32]	Cohort study	To investigate associations between serum lipoprotein subfractions and periodontitis/tooth loss	Pomerania, Germany	3,031 (47.5% male)	General adult population, excluding lipid-lowering medication users	participants not stratified by tooth loss	Excluded wisdom teeth, implants, include dentures, count by clinical exam	Conventional blood tests	No	age, sex, smoking, diabetes	Elevated triglyceride contents in LDL particles were associated with increased odds of having fewer than 20 teeth

The data fields included the following:

1. Study characteristics: Author, publication year, study design, aims
2. Participants: sample size, demographics, health conditions
3. Exposure: tooth loss classification
4. Measurement standardization: Tooth loss measurement methodology. (e.g., clinically assessed or by self-report) and cholesterol measurement methodology.
5. Confounders (e.g., age, sex)
6. Major conclusion(s)

## Results

A total of 23 studies were included in this scoping review ‘Results’ section. To clarify the findings, the studies were categorized into five groups on the basis of their method of classifying participants with tooth loss (dichotomous vs. multicategory) and the reported association with serum cholesterol levels. The results are summarized in Tables 2 and described in detail below. (Detailed information of the 23 studies listed in Table 1)

**Table 2:** Classification and key findings of 23 key studies

Group	Tooth loss classification method	Association with cholesterol	Key finding
1	Dichotomous	Related	Statistically significant associations between tooth loss and adverse changes in lipid profiles
2	Multicategory	Related	Dose-response relationship: greater tooth loss associated with worse lipid profiles (lower HDL-C, lower LDL-C and/or higher TG)
3	Dichotomous	Not related	No direct link found between tooth loss and cholesterol levels
4	Multicategory	Not related	No direct link found between tooth loss and cholesterol levels
5	Other	Varied	Described below

### Group 1: dichotomous classification with statistically significant associations

This group consists of 9 studies that stratified participants into two distinct groups. One group included five studies using a threshold of ≥ 20 remaining teeth versus < 20 remaining teeth, as retaining 20 teeth is widely considered the minimum required to maintain functional masticatory ability. Two other studies adopted a dentate versus edentulous classification, where “edentulous” refers to the complete absence of natural teeth, though denture wearers may be included. Additionally, two studies used the presence or absence of tooth loss as the grouping criterion.

Within this scoping review, five studies specifically explored the association between tooth loss and cholesterol levels, and applied the 20 remaining teeth threshold to define significant tooth loss.

After controlling for age, oral hygiene, HbA1c etc., Furukawa et al. (2007) reported a positive correlation between the number of remaining teeth and HDL-C levels in patients with type 2 diabetes [10]. In line with this result, Song et (2018) reported that participants with fewer than 20 teeth exhibited higher LDL-C levels than those with at least 20 teeth did (*p* = 0.02), although no significant difference was found in TC [11]. Apart from these two studies, a cross-sectional study by Ando et al. (2013) of 8,352 community-dwelling Japanese individuals revealed that tooth loss was significantly associated with adverse metabolic profiles, namely HDL-C [12]. Similarly, Shimizu et al. (2022) and Ono et al. (2022) also obtained consistent findings, reinforcing the overall trend [13, 14].

Collectively, the evidence indicates that tooth loss—particularly having fewer than 20 remaining teeth—is associated with dyslipidemia, characterized by reduced HDL-C and elevated LDL-C levels. This relationship is consistent across diverse populations, including general, diabetic, and elderly groups, as well as across different study designs. More importantly, the repeated use of the 20-tooth threshold further suggests that unfavorable cholesterol profiles may be linked to compromised masticatory function.

Meanwhile, 2 studies in group 1 which grouped participants based on whether they were edentulous or not, indicated that edentulism is associated with adverse lipid profiles, including elevated TC levels, lower HDL-C levels, and higher TG levels.

Johansson et al. (1994) reported that edentulous middle-aged adults exhibited significantly lower HDL-C levels (*p* < 0.05) [15]. Among women, higher TC and TG levels were also evident—differences that remained significant after adjustment for age, smoking, dietary factors etc [15]. Additionally, Oliveira et al. (2021) demonstrated that edentulism was associated with higher allostatic load over time, including unfavorable changes in HDL-C and TG levels [16].

These two studies highlight the effect of edentulism on serum cholesterol levels. Since edentulism involves the complete loss of natural teeth, it often results in reduced oral and systemic inflammation—bringing inflammatory profiles closer to those individuals with healthy oral conditions. Thus, comparisons between edentulous and dentate populations may more clearly reveal the specific impact of masticatory function loss on lipid metabolism, independent of ongoing systemic inflammatory conditions and unhealthy oral environments.

Apart from these 7 studies, two studies in group 1 stratified participants based on the presence or absence of tooth loss. Song et al. (2022) conducted a nationwide cohort study involving 65,078 South Korean participants, the study found that tooth loss was associated with an increase in TG levels [17]. Steigmann et al. (2022) conducted a study on individuals with type 1 diabetes to identify risk factors for tooth loss [18]. They found that a higher HDL-C/LDL-C ratio was significantly associated with a decreased odd of tooth loss (OR = 0.87).

These findings align with previous research indicating an association between tooth loss and cholesterol level. Moreover, these two studies provide evidence that an unfavorable cholesterol level (specifically a low HDL-C/LDL-C ratio) is a significant associated risk factor for tooth loss, indicating that there might be a mutual effect between tooth loss and cholesterol levels.

### Group 2: multicategory classification with statistically significant associations

This group consists of 7 studies that stratified participants into multiple groups based on different criteria. A dose-response relationship between the extent of tooth loss and adverse cholesterol profiles was observed.

In the study by Liljestrand et al. (2015), participants were stratified into five groups based on the number of missing teeth (0–1, 2–4, 5–8, 9–31, and 32 missing). A significant linear trend was observed wherein groups with more missing teeth exhibited progressively higher TG (p for trend = 0.031) and lower HDL-C levels (p for trend < 0.001), after adjusting for age, sex, and other cardiovascular risk factors [19].

Similarly, Chen et al. (2023) divided participants into three categories: no tooth loss (≥ 20 teeth), moderate loss (1–19 teeth), and severe loss (edentulism). They reported that HDL-C levels decreased across these groups (*p* = 0.032) [20].

Qiao et al. (2024) also used a three-category classification (0, 1–2, and ≥ 3 missing teeth) and reported a significant positive correlation between the number of missing teeth and the risk of hyperlipidemia in women (p for trend < 0.01) [21].

Furthermore, Ando et al. (2014) grouped middle-aged and elderly men into four categories (≥ 20, 10–19, 1–9, and 0 teeth) and found that the risk of dyslipidemia increased with increasing tooth loss (p for trend = 0.049) [22], further supporting a gradient effect.

Shin et al. (2017) categorized participants into three groups: 28 teeth, 20–27 teeth, and 0–19 teeth. After adjusting for confounders, a clear trend was observed for HDL-C, with mean levels decreasing as the number of teeth decreased [23].

Similarly, Zhu & Hollis (2015) classified US adults into four groups: full dentition (28 teeth), 21–27 teeth, 1–20 teeth, and edentulous. Their analysis revealed a significant positive association between the number of teeth and the HDL-C concentration (*p* = 0.003) [24]. In contrast, no significant associations were found with TG, LDL-C, or TC levels after adjustment.

Syrjälä et al. (2010) studied elderly Finns and divided them into four categories: 0, 1–9, 10–19, and 20–29 teeth. Adjusted models revealed that edentulous individuals and those with 1–9 teeth had lower mean HDL-C levels and higher mean TG levels than those with 20–29 teeth did [25].

These findings collectively underscore a consistent pattern: as the number of missing teeth increases, lipid profiles deteriorate in a stepwise manner, characterized by increasing TG levels, decreasing HDL-C levels, and elevated risks of hyperlipidemia.

### Group 3: dichotomous classification with no significant association

This group includes 4 studies that classified participants using a dichotomy. Despite the well-established association between tooth loss and serum cholesterol levels in many studies, these four articles reported no significant association between tooth loss and cholesterol levels.

Cinar et al. (2013) conducted a study among Turkish patients with type 2 diabetes. In this study, participants were dichotomized according to the mean degree of tooth loss (based on maxillary, mandibular and total), and no significant correlation was detected between tooth loss and LDL-C or HDL-C levels [26].

Furuta et al. (2016) and Taguchi et al. (2004) grouped participants based on the presence or absence of missing tooth. The former was a longitudinal study of middle-aged Japanese adults, and the latter focused on postmenopausal women. Neither of these studies revealed a significant association between tooth loss and serum cholesterol level [27, 28].

Lowe et al. (2003) examined adults in Scotland and classified participants using the dentate versus edentulous dichotomy. The results showed that cholesterol levels were not significantly linked to tooth loss after adjusting for age, social class, and smoking status confounders [29].

### Group 4: multicategory classification with no significant association

This group includes only 1 article that used the multicategory grouping method and revealed no significant association between tooth loss and serum lipid levels.

Desvarieux et al. (2003) conducted a cross-sectional analysis within the Oral Infections and Vascular Disease Epidemiology Study (INVEST) to examine the relationship between tooth loss and atherosclerosis. The study revealed only a moderate, nonlinear association between tooth loss and plaque prevalence and did not find a significant association between tooth loss and serum lipid levels (including HDL, LDL and TC) [30].

### Other studies

Gomez et al. (2022) conducted a retrospective longitudinal cohort study to assess the nutritional profile of denture wearers by tracking changes in serum biomarkers before and after receiving prosthetic treatment. No significant association was found between receiving dentures (for edentulous participants) and changes in TC, LDL, or HDL levels during the pre- versus posttreatment periods. However, patients who were partially edentulous exhibited a significant decrease in TC after they received removable partial dentures [31].

These findings suggest that prosthetic rehabilitation for partial tooth loss may help reverse a negative trend in cholesterol, possibly by restoring masticatory function and enabling a healthier diet. Nonetheless, this association was not observed in completely edentulous patients who received complete dentures. This might be because while dentures restore partial masticatory function, they may not fully replicate the chewing efficiency of natural teeth. This can lead patients to subconsciously choose for softer foods that are often lower in nutritional density. The detailed underlying is analyzed in the ‘Discussion’ section below.

Kapp et al. (2024) conducted a prospective population-based cohort study to assess the associations between serum lipoprotein subfractions and tooth loss. In this study, participants were grouped based on their baseline levels of their baseline lipoprotein levels. No significant association was found between conventional blood lipid levels (TC, LDL-C, or HDL-C) and the number of missing teeth. However, using proton nuclear magnetic resonance (^1^H-NMR) spectroscopy, a more precise profiling technique, elevated TG contents in LDL particles were associated with increased odds of having fewer than 20 teeth [32].

## Discussion

The results of this scoping review suggest an association between tooth loss and serum cholesterol levels. Most of the included studies support an association between the extent of tooth loss and adverse lipid profiles, characterized by lower HDL-C levels, higher LDL-C levels, and elevated TG levels.

### Key findings and interpretation

In this section, key findings and relative interpretations based mainly on the 23 articles which have been analyzed above are listed. Additionally, 20 relative studies will also be included to provide more evidence for these key findings and interpretations.

1. *A statistically significant association between greater tooth loss and adverse changes in serum cholesterol levels*
  Among the 23 studies that provided quantitative data on tooth loss and serum cholesterol levels, a majority (16 out of 23) confirmed a statistically significant association between greater tooth loss and adverse changes in serum cholesterol levels. This finding is further supported by additional relevant literature.
  Marina et al. (2019) identified an association between low HDL levels and tooth loss [33]. Halme et al. (2025) found that having fewer teeth is significantly associated with lower HDL-C and higher LDL-C [34]. Kim et al. (2016) and Li et al. (2009) both demonstrated that women with fewer teeth had a higher prevalence of MetS and its components, including low HDL-C [35, 36]. Furthermore, Kang et al. (2015) and Tsai et al. (2015) both found that metabolic syndrome (which includes low HDL-C in the articles) was significantly associated with having fewer than 20 teeth in women [37, 38]. Karen Raju et al. (2021) systematically reviewed evidence and concluded that HDL levels were positively correlated with the number of remaining teeth [39].
2. *Dose-response relationship between tooth loss and serum cholesterol level*
  Studies in group 2 indicate a dose-response relationship: as the number of missing teeth increases, lipid profiles deteriorate in a stepwise manner, characterized by rising TG, declining HDL-C, elevated LDL-C and elevated risks of hyperlipidemia.
3. *Distinct pathway in edentulism*
  Among the 23 key studies, the dichotomy which stratified participants into edentulous vs. dentate show that complete tooth loss (edentulism) was associated with worse lipid profiles. Wang et al. (2022) also indicates same findings [40].
  This is primarily attributed to nutritional maladaptation (e.g., reduced fiber, increased fat intake) due to impaired chewing function, rather than ongoing oral inflammation. This nutritional pathway is highlighted by Lawrence et al. (1995), who found that dentate individuals consumed a diet lower in fat and cholesterol and higher in essential nutrients compared to their edentulous counterparts [41]. Lee et al. (2004) also proposed the association between tooth loss and cholesterol levels is due to nutritional intake. They compared the cholesterol levels between white and black individuals. They found that edentulism had a more significant effect on white individuals. These findings suggest that dietary habits among white individuals may be more susceptible to the impact of edentulism, potentially due to greater dietary flexibility [42].
  These certain nutritional shifts perturb cholesterol homeostasis. Soluble fiber deficits impair bile acid binding in the gut, triggering feedback upregulation of hepatic cholesterol synthesis; concurrently, increased saturated fat intake stimulates SREBP-2-mediated LDL production [43].
  The study by Adriana et al. (2021) provides interventional support: replacing removable dentures with implant-supported fixed prostheses led to a significant reduction in cholesterol intake and an improvement in overall diet quality [44]. This study further suggesting the nutritional pathway plays an important role in the association between tooth loss and serum cholesterol levels. Meanwhile, it underscores the critical role of restoring functional chewing capacity in edentulous patients.
4. *Evidence of bidirectionality*
  Some evidence suggests the relationship may be bidirectional. In line with the result given by Steigmann et al. (2022) [18], Kikutani et al. (2004) proposed that cholesterol abnormalities might promote atherosclerosis, indirectly affecting periodontal health [45]. Marina et al. (2019) also identified dyslipidemia as a risk factor for tooth loss [33].
  These studies showed that not only does tooth loss influence cholesterol levels, but an unfavorable cholesterol profile (e.g., a low HDL/LDL ratio) was also identified as a risk factor for future tooth loss. It should be clarified that this bidirectional relationship specifically means that serum cholesterol level can be identified as a risk factor for future tooth loss, and does not imply any causal relationship.
5. *Potential for intervention*
  The study conducted by Gomez et al. (2022) discovered that prosthetic rehabilitation (e.g., removable partial dentures) for partial tooth loss was associated with an improvement inTC levels, suggesting that restoring masticatory function can positively influence metabolic health [31].
  However, both Gomez et al. (2022) and Cheraskin et al. (1969) found that even with prosthetic rehabilitation, a majority of complete denture wearers had elevated cholesterol levels, only 14.3% of whom had cholesterol level within the normal range [46]. This might due to the insufficient rehabilitation of complete dentures.
  Lee et al. (2004) revealed that although 90% of edentulous participants wore dentures, many still experienced masticatory discomfort—particularly among black individuals—indicating that dentures may not fully restore chewing function [42]. This explains for the insufficient improvement for removable dentures in edentulous participants.
  Moreover, the study by Adriana et al. (2021) suggests that implant-supported fixed prostheses improve overall diet quality, indicating the effectiveness of implant-supported fixed prostheses [44].
6. *The possible reasons why studies in group 3 and 4 found no association between tooth loss and cholesterol levels.*
  The lack of a significant association between tooth loss and cholesterol levels in these studies—contrary to the majority of the literature—may be attributed to several factors:
  A. *Primary focus on other outcomes:*
    These studies were primarily designed to investigate relationships between tooth loss and other systemic conditions (e.g., hypertension, cardiovascular disease, diabetes management, sleep apnea). Cholesterol was often a secondary outcome, and the studies may have been underpowered to detect subtle lipid changes. This reason was further suggested by the study conducted by Kapp et al. (2024) which discovered the association between tooth loss and cholesterol levels after using a more precise profiling technique [32].
  B. *Population characteristics:*
    Cinar et al. (2013) focused on diabetic patients, who already exhibit dysregulated lipid metabolism, potentially masking additional effects of tooth loss [26]. Desvarieux et al. (2003) cohort consisted of older adults (mean age 66) with a high prevalence of cardiovascular risk factors [30]. In such a population, lipid levels may be influenced more strongly by medication use, dietary habits, and metabolic conditions than by tooth loss.
  C. *Grouping methodology:*
    The use of a simple binary classification in some articles (the presence of tooth loss vs. the absence of tooth loss; dichotomized at mean) may not precisely capture the association between tooth loss and lipid changes. Other grouping method may be better. For instance: a dichotomization using the threshold of 20 remaining teeth or a multicategory classification of tooth loss may have a stronger link to serum cholesterol level.
  C. *Adjustment for confounders:*
    Some studies adjusted for variables that may be associated with tooth loss and dyslipidemia, such as dietary intake, DMFT and oral hygiene status inflammatory markers etc., this overadjustment may have attenuated the real association.
    Apart from that, confounders might also be neglected. Shen et al. (2023) found that tooth loss was associated with the severity of coronary atherosclerosis. However, cholesterol levels (TC, LDL-C, HDL-C, TG) were paradoxically lower in the moderate to severe atherosclerosis group, suggesting that medication interventions may have confounded this relationship [47]. Therefore, for some studies that did not find a correlation between tooth loss and cholesterol levels, further attention should be paid to participants’ medication use, and confounders should be more strictly controlled.
7. *Mechanisms behind the significant association.*
  A. *Nutrition pathway*
    Masticatory inefficiency reshapes dietary patterns, often reducing fiber-rich food intake while increasing consumption of processed, lipid-dense alternatives [48]. As highlighted by Dai et al. (2023) and Ishimiya et al. (2018), tooth loss can affect chewing ability, reduce dietary diversity, and induce poor oral hygiene, this may result in reduced overall nutrient intake and unhealthy dietary structures[49, 50]. This pathway is also supported by Kaumudi et al. (1996) who compared edentulous participants vs. dentate participants based on their cholesterol intake [51]. Additionally, a cross-sectional study conducted by Shino et al. (2022) demonstrate that in a frail elderly population, the combination of having few teeth and not using dentures is strongly associated with a higher prevalence of malnutrition and risk of malnutrition, as assessed by the MNA-SF [52].
  B. *Systemic inflammatory pathway*
    Tooth loss is indicative of chronic poor oral hygiene and advanced periodontal disease, which are frequently associated with sustained systemic inflammation. As demonstrated by Chen et. (2023), periodontal pathogens, such as Porphyromonas gingivalis, stimulate the activation of immune cells—including macrophages—leading to the secretion of pro-inflammatory mediators such as TNF-α, IL-6, and CRP [11, 20]. This persistent inflammatory state impairs the anti-inflammatory and antioxidant properties of high-density lipoprotein cholesterol (HDL-C), thereby diminishing its capacity to promote reverse cholesterol transport and facilitate clearance of peripheral cholesterol. These findings are further supported by the work of Ono et al. (2022) [14].
  C. *Shared risk factor pathway*
    However, the relationship is complex and confounded by overall health behaviors. Hyun et al. (2015) offer a crucial nuance: while an initial unadjusted analysis found a higher rate of tooth loss in a group with abnormal HDL-C, this association vanished after adjusting for covariates like age, gender, and health behaviors (smoking, drinking, oral hygiene) [53]. This suggests that tooth loss itself may not be an independent cause of dyslipidemia like low HDL-C. Instead, it is likely a marker for a cluster of unfavorable lifestyle factors—such as poor diet, smoking, and low health literacy—that collectively drive both poor oral health and unfavorable cholesterol levels. Therefore, the nutritional pathway operates within a broader context of shared risk factors, where tooth loss instigates dietary changes that contribute to dyslipidemia, especially when combined with other unhealthy behaviors.

### Limitations and future directions

This scoping review highlights several important limitations in the current literature on the association between tooth loss and serum cholesterol levels.

A significant constraint is that the majority of existing studies focus primarily on the relationship between tooth loss and broader systemic conditions—such as metabolic syndrome (MetS) or cardiovascular disease (CVD)—rather than directly investigating serum cholesterol levels as a primary outcome. As a result, studies reporting direct and detailed cholesterol measurements are relatively scarce.

Moreover, many included studies did not specify the methodologies used for cholesterol assay, which limits the comparability and reliability of pooled results. Future research should place greater emphasis on standardizing and transparently reporting cholesterol measurement techniques to enhance data quality and facilitate meta-analyses.

As for future studies, it is important to distinguish edentulous individuals as a distinct subgroup in further investigations. Since complete tooth loss typically eliminates active periodontal inflammation—due to the absence of periodontal diseases—edentulous populations offer a unique natural experiment to isolate the effects of masticatory functional impairment from inflammatory pathways. In these individuals, the impact of systemic inflammation or poor oral hygiene is minimized, allowing the influence of masticatory dysfunction on cholesterol metabolism to be more clearly elucidated. Thus, focused studies on edentulous populations could significantly advance our understanding of the mechanistic pathways linking oral functional loss to dyslipidemia.

To address these gaps and refine future research, several specific directions can be conducted:

1. *Edentulous vs. dentate*
  While such comparisons are common, future work must rigorously account for prosthetic status within the edentulous group (e.g., wearers of removable dentures, implant-supported prostheses, or no prostheses) and oral health status within the dentate group (e.g., number of remaining teeth, presence of periodontitis). This will help clarify whether differences in cholesterol levels are driven solely by tooth loss or are modulated by prosthetic rehabilitation and residual oral inflammation.
2. *Edentulous without removable dentures vs. edentulous with removable dentures*
  Prospective cohort studies comparing edentulous individuals with and without removable dentures would be highly valuable. Such designs could track changes in serum cholesterol levels before and after prosthetic treatment within the same individuals. Other than that, cross-sectional studies can be conducted to compare serum cholesterol level between these two groups. These studies would help determine whether restoring masticatory function through dentures—especially removable dentures—can reverse adverse lipid profiles, and to what extent.
3. *Record nutrition intake*
  Detailed dietary assessments should be integrated into future studies to explore the nutritional pathway mechanism. Recording participants’ nutrient intake—particularly fiber, saturated fat, and cholesterol consumption—would help elucidate whether observed changes in lipid profiles are mediated dietary modifications resulting from impaired chewing ability. This approach would provide direct evidence linking masticatory function to dietary behavior and subsequent metabolic outcomes.

By addressing these limitations and pursuing these targeted research directions, future studies can validate and extend this review’s findings and reveal the actual mechanism behind tooth loss and serum cholesterol levels. Moreover, these future studies would contribute to developing evidence-based interventions—such as optimized prosthetic rehabilitation—that improve oral and metabolic health.

## Data Availability

The datasets analyzed during the current study are available from the corresponding author upon reasonable request.

## Appendix

### Full search string and boolean operators

(Tooth loss OR edentulous OR edentulism) And (Cholesterol OR HDL OR LDL OR TC OR lipoprotein OR CVD OR atherosclerosis OR metabolic syndrome OR MetS).

## Abbreviations

CVD: Cardiovascular disease
HDL-C: High-density lipoprotein cholesterol
LDL-C: Low-density lipoprotein cholesterol
INVEST: Oral Infections and Vascular Disease Epidemiology Study
MetS: Metabolic syndrome
MNA-SF: Mini Nutritional Assessment – Short Form
OR: Odds ratio
PRISMA-ScR: Preferred Reporting Items for Systematic reviews and Meta-Analyses extension for Scoping Reviews
TC: Total cholesterol
TG: Triglycerides
TNF-α: Tumor necrosis factor alpha
IL-6: Interleukin-6
CRP: C-reactive protein
SREBP-2: Sterol regulatory element-binding protein 2
^1^H-NMR: Proton nuclear magnetic resonance
